# Butyrate suppresses atherosclerotic inflammation by regulating macrophages and polarization via GPR43/HDAC-miRNAs axis in *ApoE*^−/−^ mice

**DOI:** 10.1371/journal.pone.0282685

**Published:** 2023-03-08

**Authors:** Huiyan Ma, Libo Yang, Yajuan Liu, Ru Yan, Rui Wang, Peng Zhang, Zhixia Bai, Yuanyuan Liu, Yi Ren, Yiwei Li, Xin Jiang, Ting Wang, Ping Ma, Qining Zhang, Aifei Li, Mixue Guo, Xiaoxia Zhang, Shaobin Jia, Hao Wang

**Affiliations:** 1 Clinical Medical College, Ningxia Medical University, Yinchuan, China; 2 Heart Centre & Department of Cardiovascular Diseases, General Hospital of Ningxia Medical University, Yinchuan, China; 3 Ningxia Key Laboratory of Vascular Injury and Repair Research, Ningxia Medical University, Yinchuan, China; 4 School of Basic Medical Sciences, Ningxia Medical University, Yinchuan, China; 5 College of Traditional Chinese Medicine, Ningxia Medical University, Yinchuan, China; Max Delbruck Centrum fur Molekulare Medizin Berlin Buch, GERMANY

## Abstract

Chronic low-grade inflammation is regarded to an important signature of atherosclerosis (AS). Macrophage (Mψ) and related polarization have been demonstrated to play a crucial role in the occurrence and development of AS inflammation. Butyrate, a bioactive molecule produced by the intestinal flora, has been increasingly demonstrated to exhibit a vital role for regulating the inflammation in chronic metabolic diseases. However, the effectiveness and multiple anti-inflammation mechanisms of butyrate on AS still need to be further understood. *ApoE*^*−/−*^ mice fed with high-fat diet as AS model were administered with sodium butyrate (NaB) for 14 weeks of treatment. Our results showed that the atherosclerotic lesion in the AS group was dramatically reduced after NaB intervention. Moreover, deteriorated routine parameters of AS including body weights (BWs), low-density lipoprotein (LDL-C), triglyceride (TG), total cholesterol (TC) were significantly reversed by NaB administration. Abnormal elevated plasma and aorta pro-inflammatory indicators including interleukin (IL)-1β, IL-6, IL-17A, tumor necrosis factor (TNF)-α and lipopolysaccharide (LPS), as well as reduced anti-inflammatory IL-10 in plasma were respectively rectified after NaB administration. Consistently, accumulated Mψ and associated imbalance of polarization in the arota were attenuated with NaB treatment. Importantly, we demonstrated that the suppression of Mψ and associated polarization of NaB was dependent on binding G-protein coupled receptor (GPR) and inhibiting histone deacetylase HDAC3. Moreover, we found that intestinal butyrate-producing bacteria, anti-inflammatory bacteria and intestinal tight junction protein zonula occludens-1 (ZO)-1 may contribute to this effectiveness. Intriguingly, according to transcriptome sequencing of atherosclerotic aorta, 29 elevated and 24 reduced miRNAs were found after NaB treatment, especially miR-7a-5p, suggesting that non-coding RNA may possess a potential role in the protection of NaB against AS. Correlation analysis showed that there were close complicated interactions among gut microbiota, inflammation and differential miRNAs. Collectively, this study revealed that dietary NaB may ameliorate atherosclerotic inflammation by regulating Mψ polarization via GPR43/HDAC-miRNAs axis in *ApoE*^−/−^ mice.

## Introduction

Atherosclerosis (AS) represents a basis of cardiovascular diseases with the highest morbidity and mortality worldwide [1], of which the pathological features are mainly described as inflammation and lipid metabolism disturbance [2]. Vascular intimal macrophages (Mψs) are thought to be responsible for the maintenance of atherosclerotic inflammation in the development of AS [3].

Numerous studies have shown that Mψs and associated polarization play a vital role in the initiation and development of AS inflammation [4–6]. M1 Mψ is mainly involved in the promotion of inflammatory response. In contrast, M2 Mψ shows anti-inflammation effect and promotes tissue repair by producing anti-inflammatory factors such as interleukin (IL)-10. In the pathological conditions, accumulating Mψs along with subsequently polarization to M1, are responsible for aggravating AS inflammation [7]. Conversely, M2 Mψ are thought to secret anti-inflammatory IL-10 and collagen, contributing to the stability of AS plaques [8]. Thus, the regulation of Mψ and associated M1/M2 polarization may be a potential therapeutic strategy for AS.

Accumulating evidence suggests that gut dysbiosis is closely associated with exacerbation of AS progression [9, 10]. The formation and rupture of atherosclerotic plaque are related to the high levels of gut microbiota-derived lipopolysaccharide (LPS) and inflammatory cytokines in the circulation [11]. Short-chain fatty acids (SCFAs), mainly acetate, propionate and butyrate, are the main end-products of the bacterial fermentation of nondigestible dietary fibers within the lumen of the mammalian colon [12]. Compared to other SCFAs, emerging studies have demonstrated that butyrate presents an anti-inflammatory effect in chronic metabolic diseases [13, 14]. Butyrate and related butyrate-producing bacteria are inversely correlated with atherosclerotic lesion [15–17].

Butyrate exerts an anti-inflammatory effect mainly by both binding to G protein-coupled receptors (GPRs) and inhibiting histone deacetylases (HDACs) [12]. Butyrate-binding receptors, GPRs, which mainly include GPR41, GPR43, and GPR109a, can suppress the recruitment of inflammatory cells and the production of pro-inflammatory cytokines. As a major butyrate receptor, GPR43 highly expressed in Mψ may mediate anti-inflammatory response [16]. Studies from animal models have confirmed that sodium butyrate could suppress the activation of nuclear factor-κB (NF-κB) pathway via GPR43 and β-arrestin-2 to inhibit inflammation [15, 18, 19]. Peroxisome proliferator-activated receptors (PPARs) are ligand-activated transcription factors that exert significant impacts on metabolism-related pathways [20]. The elevation of PPARγ by butyrate was involved in the inhibition of NF-κB pathway, resulting in the improvement of inflammation [21]. Butyrate also affects the rate of neointima formation by reducing the activation of Nod-like receptor pyrin domain 3 (NLRP3) inflammasome [22]. Transcription factor specificity protein 1 (Sp1) is often compounded with histone deacetylases (HDACs) to regulate acetylation of target genes. Notably, butyrate as an inhibitor of HDACs influence the formation of the Sp1/HDAC complex [23].

MicroRNAs (miRNAs) play an critical role in the regulation of the development of AS [24]. MiRNAs are well-known for regulating gene expression at the post-transcriptional level by pairing with target sequences in the 3′ untranslated region of mRNAs. The endogenously expressed miRNAs play important roles in many physiological and pathological processes [25]. MiR-205 was epigenetically regulated by HDAC2 through an Sp1-mediated pathway [25]. MiR-7a/b protected against cardiac remodeling and hypoxia-induced injury in H9c2 cardiomyoblasts involving Sp1 and PARP-1 [26]. The bulk of evidence has demonstrated the central role of epigenetic machinery in Mψ polarization [27].

In the present study, we examined the effects of orally administered butyrate on AS progression and associated mechanisms including Mψ polarization in inflammation, gut microbiota, and the role of miRNAs in HFD-induced atherosclerotic Apolipoprotein E deficiency (*ApoE*^−/−^) mice.

## Materials and methods

### Animal experiments

All animal protocols used in this study were approved by the Ethics Committee of Ningxia Medical University (No. 2020–527). Thirty male Jackson (C57BL/6J) mice aged 8 weeks and weighing 18–22 g were purchased from Ningxia Medical Laboratory Animal Center. Thirty male *ApoE*^*−/−*^ mice (8-week-old) were obtained from Vital River Laboratory Animal Technology Co., Ltd., Beijing, China. All the mice were maintained under standard, specific, and pathogen-free conditions in individual cages in a temperature-controlled room (ambient temperature 22 ± 1°C, air humidity 40–70%) with a 12 h light/dark cycle in Ningxia Medical Laboratory Animal Center. A high-fat diet (HFD) with 0.5% cholesterol (No. TP28520) was purchased from TROPHIC Animal Feed High-tech Co., Ltd., Nantong, China. The exact product description of HFD and normal diet were supported in S1 Table. Sodium butyrate (NaB, purity>98, No. V900464) was obtained from Sigma (St Louis, MO, USA).

### Experimental design

**Fig 1:**
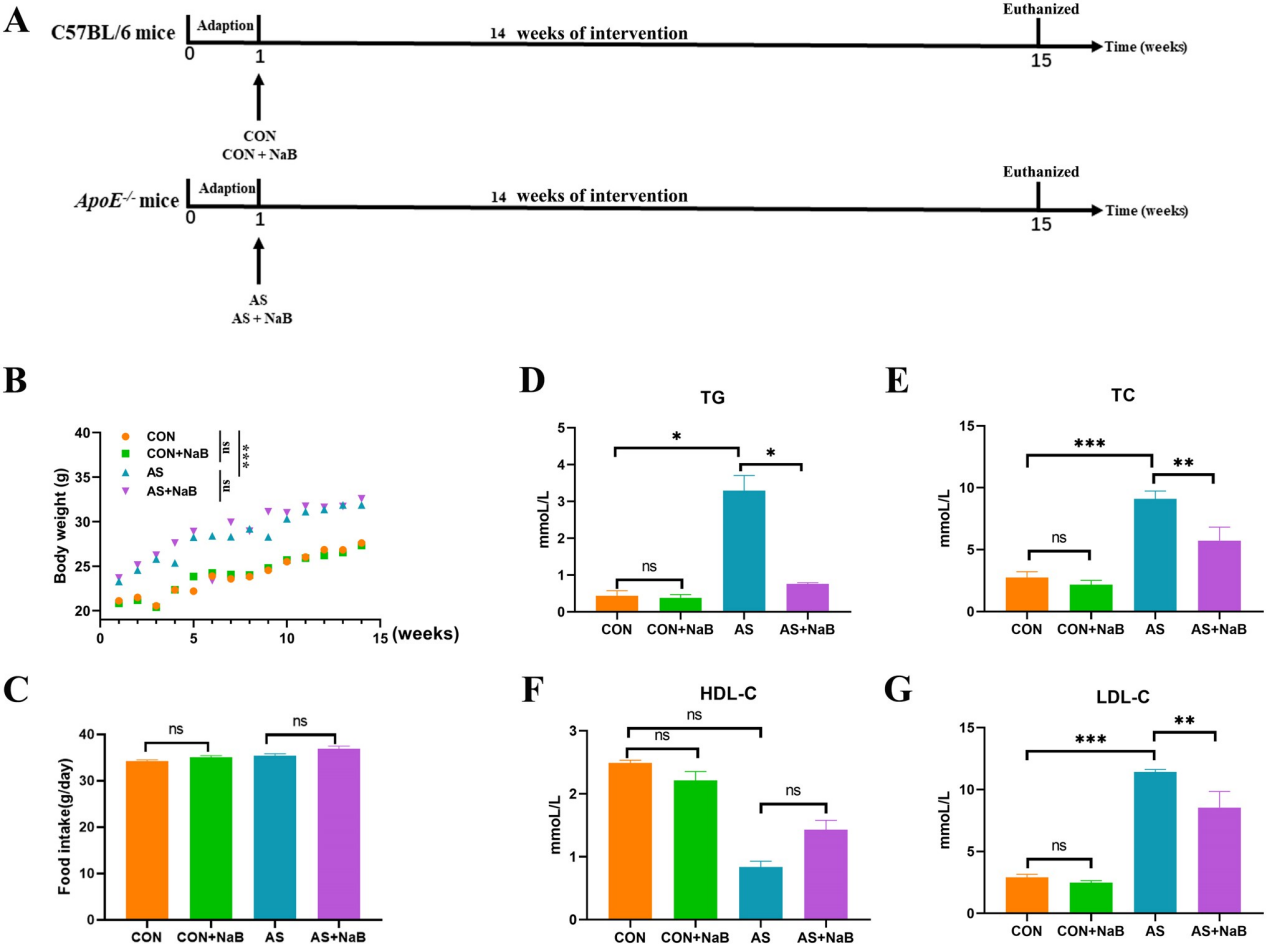
NaB alleviated physiological parameters and serum lipids in diverse groups. (A) Schematic time diagram of the experimental design. (B) Body weights (BWs) of 4 groups. (C) Food intake. (D) Triglyceride (TG). (E) Total cholesterol (TC). (F) High-density lipoprotein (HDL-C). (G) Low-density lipoprotein (LDL-C). Data were expressed as mean ± SEM. *P<0.05, **P<0.01, ***P<0.001. ns, no significant difference. CON: control group; CON+NaB: sodium butyrate (NaB)-fed control group; AS: model group; AS+NaB: NaB-fed model group.

As shown in Fig 1A, after one week of adaption the mice were randomly assigned to 4 groups (n = 15/each group): control group (CON), CON treated with NaB group (CON+NaB), atherosclerosis group (AS) and AS treated with NaB group (AS+NaB). C57BL/6J mice in the CON or *ApoE*^*−/−*^ mice in AS were respectively fed normal or HFD diet. Meanwhile, mice in CON and AS groups were administered normal saline, as well as mice in CON+NaB and AS+NaB groups were fed with NaB (200mg/kg, dissolved by normal saline) by gavage once daily. During the experiment, body weights (BWs) were monitored weekly and food intake was recorded every 2 days. After 14 weeks of feeding, stool samples were freshly obtained and immediately frozen at −80°C for the subsequent analysis. All mice were euthanized with 4% sodium pentobarbital and associated indications were investigated. Blood samples were rapidly collected by orbital bleeding and centrifuged at 4°C (1,200 × g for 15 min) to obtain plasma samples, which were stored at −80°C for further study.

### Histology and morphometry evaluations of atherosclerotic lesions

The pathological changes in AS were measured with *en face* oil red O staining, HE staining, and Masson’s trichrome staining. Images captured with Canon EOS 70D camera were analyzed using Image J 8.0 software (National Institutes of Health, United States). The lesion area index was calculated as the percentage of aortic lumen area covered by atherosclerotic lesions. The necrotic core was measured by Image-Pro plus software, and the ratio of the positive area to the total plaque area was calculated for statistical analysis as previously described [28]. Observers were blinded to the experimental groups.

### Flow cytometry

Mψs, the significant aortic inflammatory cells, were isolated from aortic tissues. Briefly, 1 g of aortic tissues was minced and suspended in 5 ml of Hanks balanced salt solution (HBSS) containing 0.1% (w/v) collagenase type IV (Sigma, United States) for 20 min at 37°C. Next, the specimen was washed with RPMI1640 containing 2% of fetal bovine serum (FBS) and then filtered through a 200-mesh nylon membrane. After centrifugation at 70 × g for 3 min at 4°C, the supernatants were discarded, and the pellets were resuspended in 3 ml HBSS. After the erythrocyte lysis, samples were centrifuged for 5 min at 500 × g, 4°C, and then washed 2 times. The final concentration was adjusted to 1 × 10^7^ cells/ml. To stain Mψs, 1 μl of PE-anti-F4/80 antibody, APC-anti-iNOS antibody, BP450-anti-CD206 antibody, and FITC-anti-TLR4 antibody (Biolegend, United States) were simultaneously added in 100 μl of cell suspension and incubated on the ice in the dark for 30 min. The prepared samples were measured and analyzed using the Beckman Cyto FLEX flow cytometer (Beckman Bioscience, United States).

### Plasma LPS assay

The plasma LPS level in each group was examined using a Limulus amebocyte lysate kit (Xiamen Bioendo Technology Co., Ltd., Xiamen, China) according to the manufacturer’s instruction. Briefly, the plasma was diluted with endotoxin-free water (1:4). Then 50 μl of diluted plasma was put into each well in a 96-well plate. At the initial time point, 50 μl of the Limulus amebocyte lysate reagent was added to each well. The plate was incubated at 37°C for 30 min. Then, 100 μl of chromogenic substrate warmed to 37°C was added to each well, and the incubation was extended for an additional 6 min at 37°C. Finally, the reaction was stopped by adding 100 μl of 25% solution of glacial acetic acid. Optical density at 545 nm was measured with a microplate reader (Thermo Scientific, United States).

### Inflammatory cytokines

Tumor necrosis factor (TNF)-α, interferon (IFN)-γ, IL-6, IL-1β, IL-17A, and IL-10 were respectively determined by RayBiotech (QAM-INT-1-1) chips (Quantibody^®^ Mouse Interleukin Array) according to the manufacturer’s instructions.

### Measurements of plasma lipid profiles

Plasma levels of triglycerides (TG), total cholesterol (TC), high-density lipoprotein (HDL-C) and low-density lipoprotein (LDL-C) were measured by an automatic biochemical analyzer (AU400 Olympus, Japan).

### Quantitative real-time PCR

Transcriptional mRNA levels of genes were performed by quantitative real-time PCR (qRT-PCR). After RNA was isolated from the aorta tissue, cDNA was synthesized using M-ML V reverse transcriptase (Invitrogen; Thermo Fisher Scientific, Inc.) according to the manufacturer’s instructions. qPCR (ABI VII7 PCR System, Applied Biosystems; Thermo Fisher Scientific, Inc.) was conducted in a 20 μl reaction volume (10 μl SYBR Green Master Mix, 0.8 μl PCR Forward Primer (10 μM), 0.8 μl PCR Reverse Primer (10 μM), 0.4 μl ROX, 2 μl cDNA, and 6 μl nuclease-free water) with the following protocol: initiation at 95°C for 5 min, followed by 40 cycles of 95°C (5 sec) and 60°C (34 sec). GAPDH was used as a reference. The assay was performed in three replicate wells, and three parallel experiments for each sample were conducted. The 2-^ΔΔCt^ methods were used to calculate relative RNA expression levels. Primers sequences were presented in S2 Table.

### Gut microbiota analysis

The mice in each group were transferred to fresh and sterilized cages after 14 weeks of treatment. The fresh feces of each group were individually collected and immediately frozen into liquid nitrogen, finally stored at −80°C until the DNA extraction. Cetyltrimethylammonium bromide (CTAB) method [29] was used to extract the genomic DNA of samples, and then the purity and concentration of the DNA were detected by agarose gel electrophoresis and Nanodrop one (Thermo Fisher, USA). Briefly, 16S rRNA genes were amplified by using V3-V4 regions bacterial primers (341F 5’- CCTAYGGGRBGCASCAG-3’ and 806R 5’- GG ACTACNNGGGTATCTAAT-3’). All PCR reactions were carried out with Phusion1 HighFidelity PCR Master Mix (New England Biolabs, USA). Sequencing libraries were generated using the Ion Plus Fragment Library Kit 48 rxns (Thermo Scientific, USA). The library quality was assessed on the Qubit^@^ 2.0 Fluorometer (Thermo Scientific, USA). The library was sequenced on an Illumina HiSeq 2500 platform (Illumina, USA) by Beijing Nuo He Zhi Yuan Technology Co., Ltd., China.

### Transcriptome sequencing and analysis

Whole aorta transcriptome library preparation and deep sequencing were conducted by Biomarker Technologies Co, Ltd. The purity was determined using an ultra-microspectrophotometer (optical density 260 nm, NanoDrop; Thermo Fisher Scientific, Inc.), n = 3/group. DESeq R package was used to identify the significantly dysregulated miRNAs with cut-off criteria: P<0.05 and |log2 fold change|>1.

### GO and KEGG pathway analysis

To better understand the biological functions and potential mechanisms of miRNAs in the effectiveness of NaB on AS, GO enrichment and KEGG pathway analyses were employed on these predicted target genes of differentially expressed miRNAs. Briefly, GO analyses (www.geneontology.org) consisted of three components: biological process (BP), cellular component (CC), and molecular function (MF). KEGG analyses were carried out to investigate the potential significant pathways (http://www.genome.jp/kegg/).

### Statistical analysis

The data shown as the mean ± SEM were conducted with Prism 8.01 (GraphPad Software Inc., CA, United States). Two-way analysis of variance (ANOVA) followed by the Turkey multiple-comparison test was used to determine statistical difference between experimental groups. Correlation analysis was performed using the Spearman method. P<0.05 was considered statistically significant.

## Results

### NaB alleviated physiological parameters and serum lipids

To assess whether the difference in diet intake contributes to the effects of NaB treatment, food consumption and body weights were monitored. The body weights in AS group showed a steady weight gain and subsequently increased after 14 weeks, compared to the CON group (Fig 1B). NaB treatment showed no effect on weight gain compared to AS group. In terms of food intake, the average intake of mice in each group was decreased during the intervention period, but without a significant difference (Fig 1C), suggesting that NaB administration showed no influence in energy intake.

After 14 weeks of treatment, the serum biochemical parameters of mice were respectively determined. Compared to the CON group, plasma levels of TG (P<0.05; Fig 1D), TC (P<0.001; Fig 1E), and LDL-C (P<0.001; Fig 1G) in AS group were notably increased. After NaB administration, plasma TC, TG and LDL-C levels were significantly rectified (Fig 1D, 1E and 1G). A decrease trend of HDL-C (Fig 1F) in plasma was observed in AS group without significant difference compared with CON group. It also showed no significant difference in HDL-C level in supplementary NaB group during HFD. Moreover, there was no significant difference in serum lipids between CON and CON+NaB groups. Collectively, these data demonstrated that NaB could protect against dyslipidemia in atherosclerotic *ApoE*^−/−^ mice.

### NaB consumption ameliorated atherosclerosis

**Fig 2:**
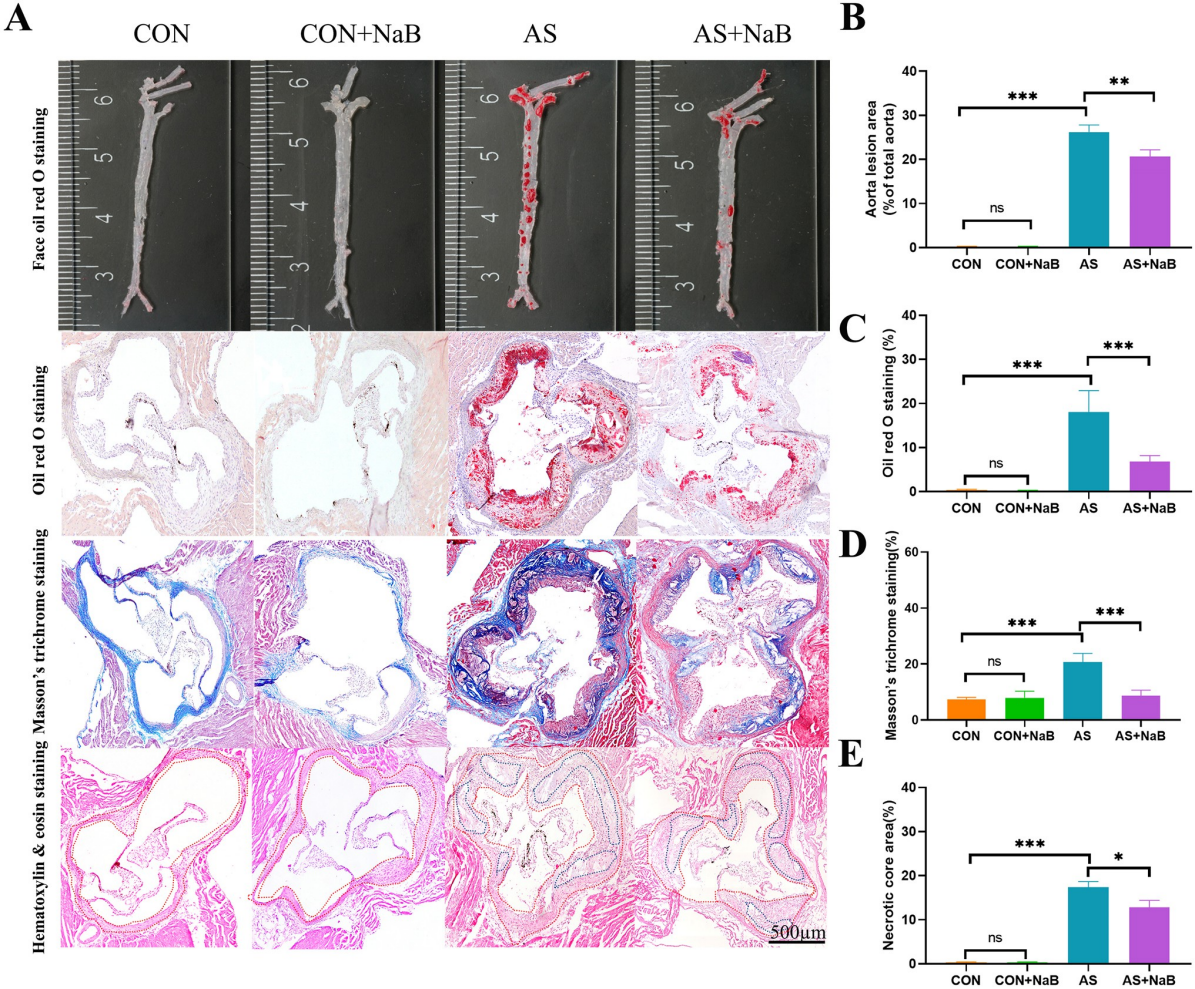
NaB consumption reduced aorta atherosclerosis. (A) Representative sections of the valve area of the aortic root of the heart were stained with *en face* oil red O staining, oil red O staining, Masson’s trichrome staining and hematoxylin&eosin staining, respectively, Quantitative analysis as lesion area/total area (%). (B) face oil red O staining. (C) oil red O staining. (D) Masson’s trichrome staining and (E) Relative necrotic core area expressed as percentage of the total plaque area. *P<0.05, **P<0.01, ***P<0.001. ns, no significant difference. The bar of 500 μm was presented in the right corner of (A).

To further elucidate the involvement of NaB in the amelioration of atherosclerosis, both *en face* analyses of the aorta and the cross-sectional analyses of the aortic sinus area were evaluated. Histopathologic staining including *en face* oil red O staining, oil red O staining, Masson’s trichrome staining and HE staining were used to measure atherosclerotic plaque, fibrosis, and pathological damage in the aortic root of the heart, respectively. As shown in Fig 2A, lesion area and necrotic core size in the aortic sinus were remarkably exacerbated in AS mice. The percentage of *en face* oil red O staining in the AS group was notably higher than that in the CON group (P<0.001; Fig 2B). Similar aggregated results of oil red O staining (P<0.01) and Masson’s trichrome staining (P<0.001) were separately observed in AS model, compared to the CON group (Fig 2C and 2D). In addition, H&E staining of the aortic sinus revealed a significant increase in necrotic core size in the AS group (Fig 2E). After intervention with NaB, the necrotic core size was decreased significantly. Intriguingly, these pathological lesions in AS were attenuated with NaB administration (P<0.05; Fig 2E). In addition, no lesion was found between the CON group and CON+NaB group (Fig 2B, 2C and 2D). Taken together, these results demonstrated that NaB intervention could ameliorate the atherosclerotic lesions.

### Dietary NaB significantly reduced chronic inflammation in AS

**Fig 3:**
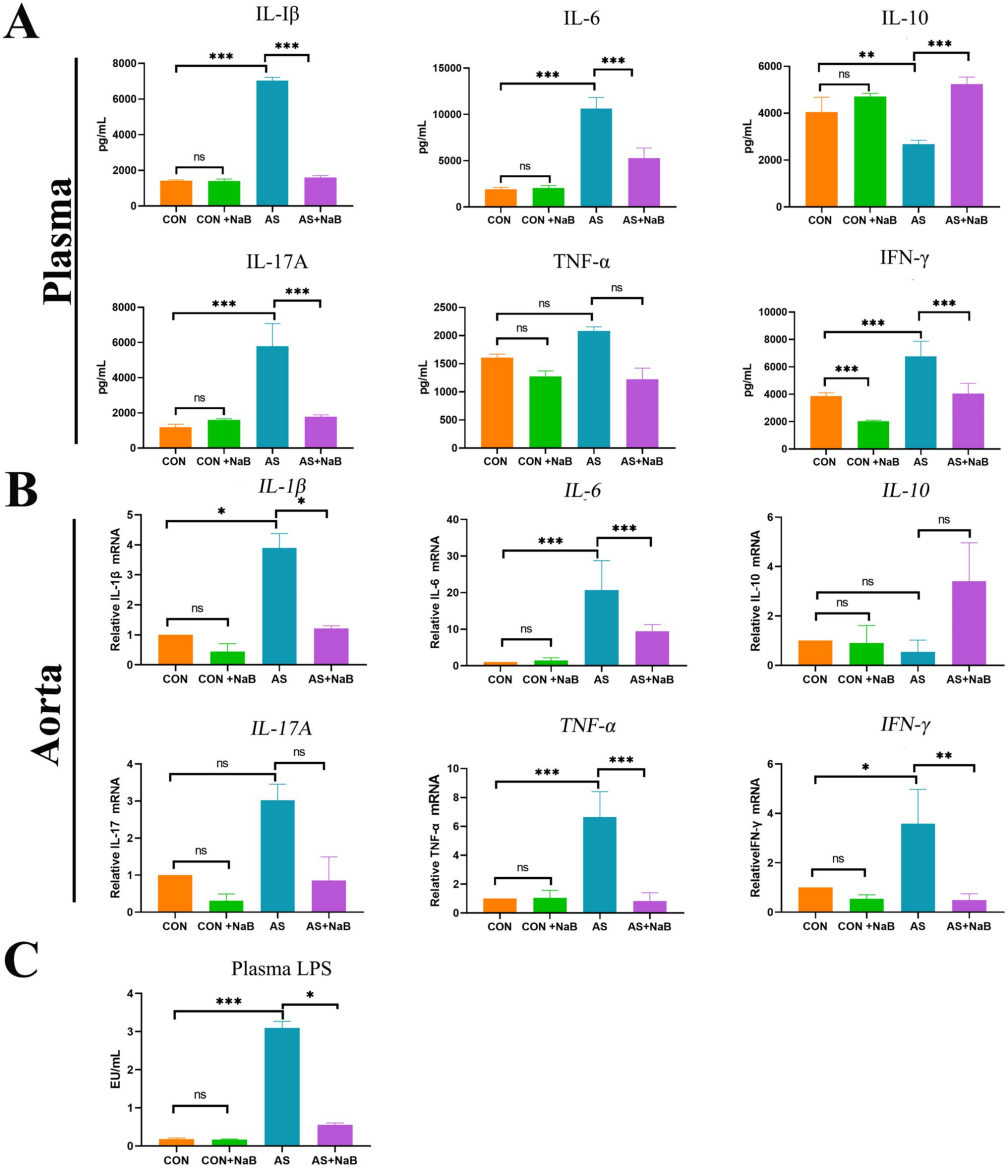
Dietary NaB significantly reduced chronic inflammation in AS. (A) Plasma of mice from 4 groups were respectively collected for the determination of interleukin (IL)-1β, IL-6, IL-17A, IL-10, tumor necrosis factor (TNF)-α, interferon (IFN)-γ by RayBiotech chip detection. (B) RT-PCR was used to determine relative mRNA levels of IL-1β, IL-6, IL-17A, IL-10, TNF-a, and IFN-γ in aorta tissues. (C) Plasma lipopolysaccharide (LPS) levels in diverse groups were measured using a Limulus amebocyte lysate kit. *P<0.05, **P<0.01, ***P<0.001. ns, no significant difference.

Mounting scientific proofs over decades have suggested that atherosclerosis represents a chronic inflammatory disorder [30]. Accumulating evidences support that NaB possesses the ability to reduce the expressions of pro-inflammatory cytokines [31]. We further examined concentrations of pro-inflammatory cytokines including IL-1β, IL-6, IL-17A, TNF-α, IFN-γ, as well as anti-inflammatory IL-10 (Fig 3A), respectively. The results showed that plasma levels of pro-inflammatory IL-1β, IL-6, IL-17A and IFN-γ in the AS group were significantly increased compared to the CON group, but the anti-inflammatory IL-10 was notably decreased. After the dietary NaB intervention, the concentrations of IL-1β, IL-6, IL-17A and IFN-γ in plasma were decreased and anti-inflammatory IL-10 was increased compared with those in the AS group.

In parallel, mRNA levels of *in situ* aortic inflammatory TNF-α, IL-1β, IL-6 IL-17A, IFN-γ, and IL-10 were determined to evaluate the effects of dietary NaB on plaque inflammation in atherosclerotic mice (Fig 3B). Similar to the above plasma levels of inflammation, aggravated TNF-α, IL-1β, IL-6, IFN-γ (all P<0.05) in aortic tissues were remarkably decreased after dietary NaB intervention. Moreover, a decrease trend of anti-inflammatory IL-10 in aorta was observed in AS group without significant difference compared to CON group, which also showed no significant difference after NaB treatment. These results suggested that dietary NaB treatment ameliorated the inflammation in atherosclerotic *ApoE*^−/−^ mice.

### Dietary NaB reduced plasma LPS levels

LPS-mediated inflammation based on gut-heart axis has been thought to contribute to the AS aggravation (29). Thus, we further tested the plasma LPS in AS. Plasma LPS levels in AS group were higher than those in the CON group (P<0.001; Fig 3C), which was significantly decreased after NaB intervention (P<0.05; Fig 3C), demonstrating that dietary NaB may ameliorate LPS-induced intestinal barrier dysfunction and subsequent translocated circulating endotoxemia.

### NaB regulated atherosclerotic Mψs and M1/M2 polarization in mice

**Fig 4:**
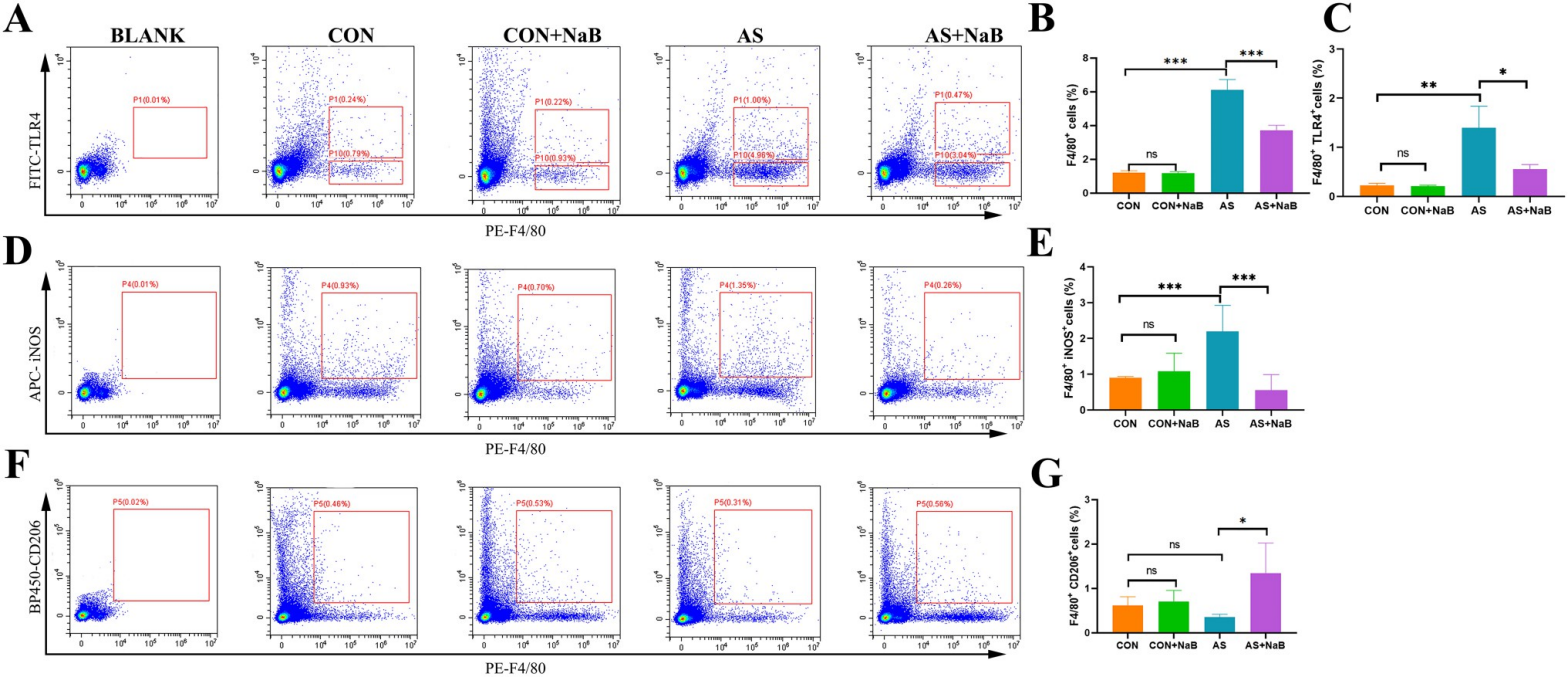
Effects of NaB on Mψ and associated polarization in *ApoE*^−^/− mice with AS by flow cytometry. (A) Flow cytometry analysis of aorta F4/80^+^TLR4^+^ Mψs in diverse groups. (B) The proportion of aorta F4/80^+^ Mψs. (C) The proportion of aorta F4/80^+^ TLR4^+^ Mψs. (D) Flow cytometry analysis of aorta F4/80^+^ iNOS^+^ M1 Mψs in diverse groups. (E) The proportion of aorta F4/80^+^ iNOS^+^ M1 Mψs. (F) Flow cytometry analysis of aorta F4/80^+^ CD206^+^ M2 Mψs in diverse groups. (G) The proportion of aorta F4/80^+^ CD206^+^ M2 Mψs. *P<0.05, **P<0.01, ***P<0.001. ns, no significant difference. All experiments were performed in triplicate. Mψ: macrophage; TLR4: Toll-like receptor 4.

Mψs and associated polarization play a vital role in the formation and progression of atherosclerotic lesions [32]. To further analyze the effects of NaB on aortic Mψs, aortic F4/80^+^TLR4^+^ Mψs were measured by flow cytometry (Fig 4A). The ratios of aortic F4/80^+^ cells and F4/80^+^TLR4^+^ cells were increased in AS group compared to CON group (P<0.01; Fig 4B and 4C). However, the proportions of F4/80^+^ TLR4^+^ cells and F4/80^+^ cells were respectively lower after NaB treatment (P<0.05; Fig 4B and 4C). As shown in Fig 4D, iNOS, M1 Mψs-associated marker, in aortic plaques of model group was increased significantly (P<0.05; Fig 4E) compared with control group, and exhibited an obvious decrease with NaB treatment. Conversely, the expression of M2 Mψs marker CD206 in aorta of model group after dietary NaB intervention was elevated (Fig 4F and 4G), suggesting that NaB may alleviate AS via regulating total Mψs and polarization by suppressing M1 polarization and enhancing M2 activation.

### NaB inhibited inflammation via GPR43 and HDAC mediated pathways

**Fig 5:**
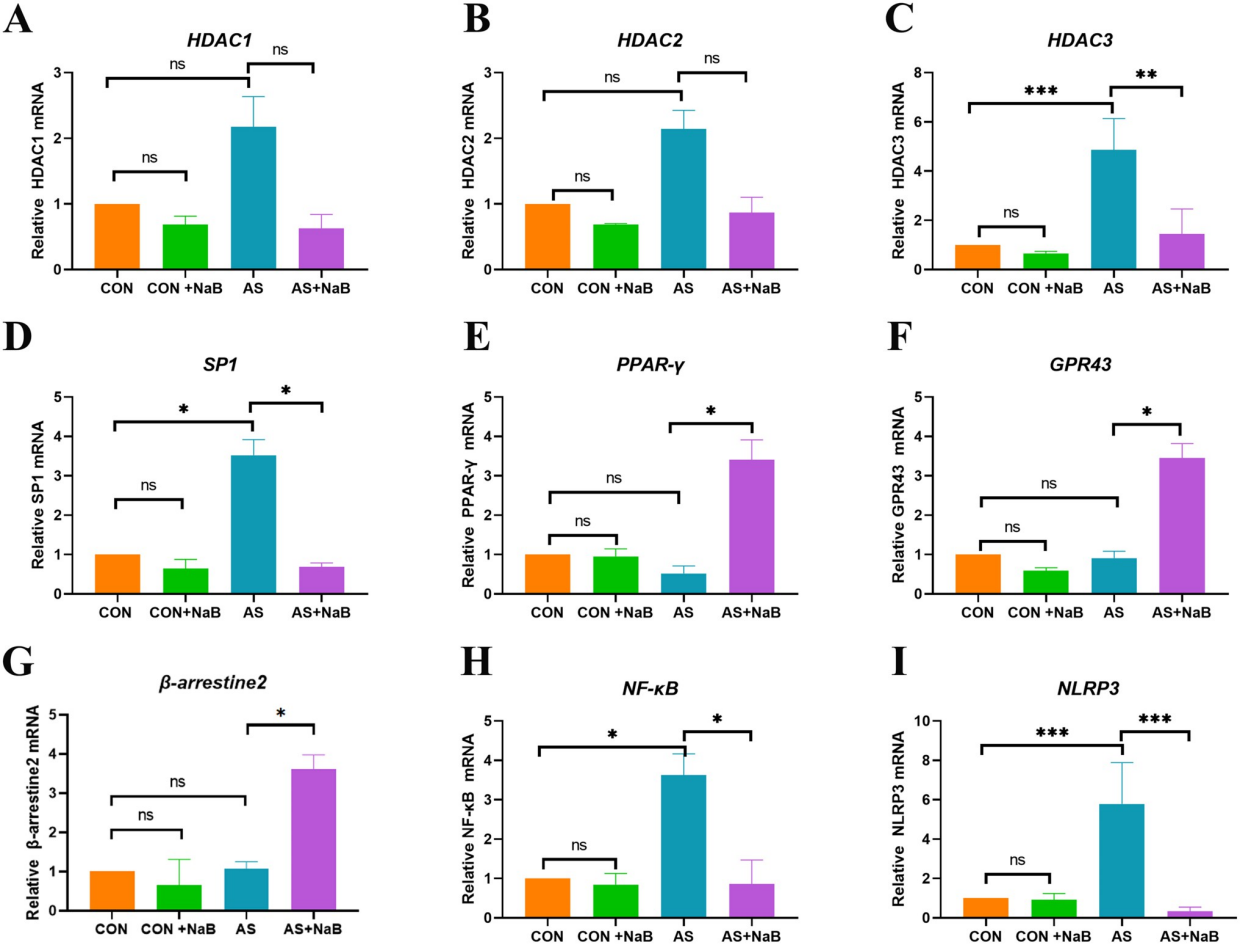
NaB inhibited inflammation via GPR43 and HDAC mediated pathway. RT-PCR was used to determine relative mRNA levels of HDAC1 (A); HDAC2 (B); HADC3 (C); Sp1 (D); PPAR-γ (E); GPR43 (F); β-arrestin-2 G); NF-κB (H) and NLRP3 (I) in aorta tissues. *P<0.05, **P<0.01, ***P<0.001. ns, no significant difference.

NaB exerts pleiotropic biological effects mainly by activating G protein-coupled receptors (GPRs) and inhibiting histone deacetylases (HDACs) [33]. As shown in Fig 5, compared with HFD-fed mice, NaB treatment elevated the transcriptional levels of GPR43, PPAR-γ, and β-arrestin-2, as well as down-regulated HDAC3, Sp1, NF-κB, and NLRP3. These results indicated that GPR43 and HDAC signaling pathway may probably involve in NaB-mediated regulation of inflammation in the atherosclerotic *ApoE*^−/−^ mice.

### NaB consumption enriched intestinal butyrate-producing and anti-inflammatory bacteria

Growing evidence has demonstrated that gut dysbiosis is closely associated with the development of AS [29, 34–37]. To further confirm the effects of NaB on gut microbiota in diverse groups, bacterial community were investigated by 16S rRNA sequencing and analysis.

**Fig 6:**
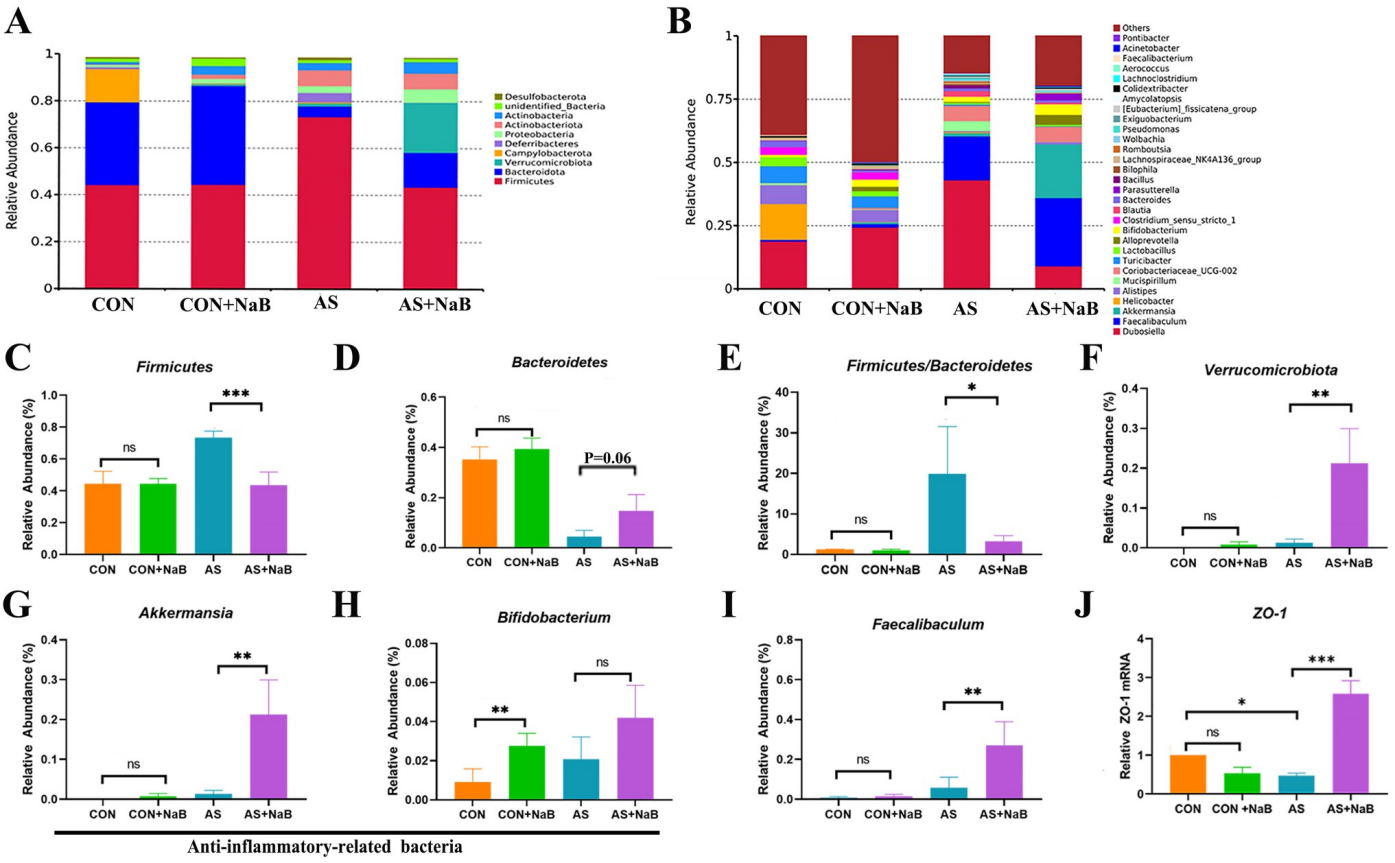
NaB consumption modulated the composition of gut microbiota. (A-I) The phylum and the genus levels. (J) The mRNA expression level of ZO-1 in different groups. *P<0.05, **P<0.01, ***P<0.001. ns, no significant difference.

At the phylum level, microbial composition of all mice was dominated with *Firmicutes* (CON 44%, CON+NaB 45%, AS 73%, AS+NaB 43%) and *Bacteroidetes* (CON 35%, CON+NaB 42%, AS 0.05%, AS+NaB 15%) (Fig 6A). We found an obviously decreased abundance of *Firmicutes* (P< 0.001) and an increase trend of *Bacteroidetes* (P = 0.06) in AS+NaB group compared to the AS group (Fig 6C and 6D). The ratio of *Firmicutes/Bacteroidetes* (F/B) was increased (P<0.05; Fig 6E) in the AS group, which was reversely decreased after the intervention of dietary NaB (P<0.05; Fig 6E). Thus, the NaB had a major influence on the F/B ratio under the HFD feeding in the atherosclerotic mice. In addition, NaB also restored the increased abundance of *Verrucomicrobiota* in AS (P<0.01; Fig 6F).

The relative abundance of microbiota at the genus level was shown in Fig 6B. The relative abundance of *Akkermansia* and *Faecalibaculum* in the AS+NaB group were significantly increased compared to the AS group (all P<0.01, Fig 6G and 6I). Additionally, *Bifidobacterium* in the CON+ NaB group was higher than those in the CON group (P<0.01; Fig 6H). In brief, the data summarized here clearly indicated that exogenous butyrate altered the composition of the microbiota in AS. Importantly, butyrate-producing bacteria *Faecalibaculum* was increased in the AS+ NaB group compared to the AS group (Fig 6I). The above-mentioned results indicated that butyrate reduced atherosclerosis development by regulating butyrate-producing bacteria of gut microbiota.

To further assess the integrity of gut mucosal barrier after the above rectification of gut dysbiosis with NaB treatment, tight junction protein ZO-1 expression in diverse groups was determined (Fig 6J). Compared to the CON group, intestinal ZO-1 expression in AS group was significantly reduced, indicating that the integrity of gut mucosa was impaired in AS. However, gut mucosal ZO-1 level of AS mice showed a notable elevation after the supplementation with NaB, demonstrating that NaB administration may contribute to enhancing the integrity of the gut barrier (P<0.001; Fig 6J).

### Correlation analysis among gut microbiota, inflammation and serum lipids

**Fig 7:**
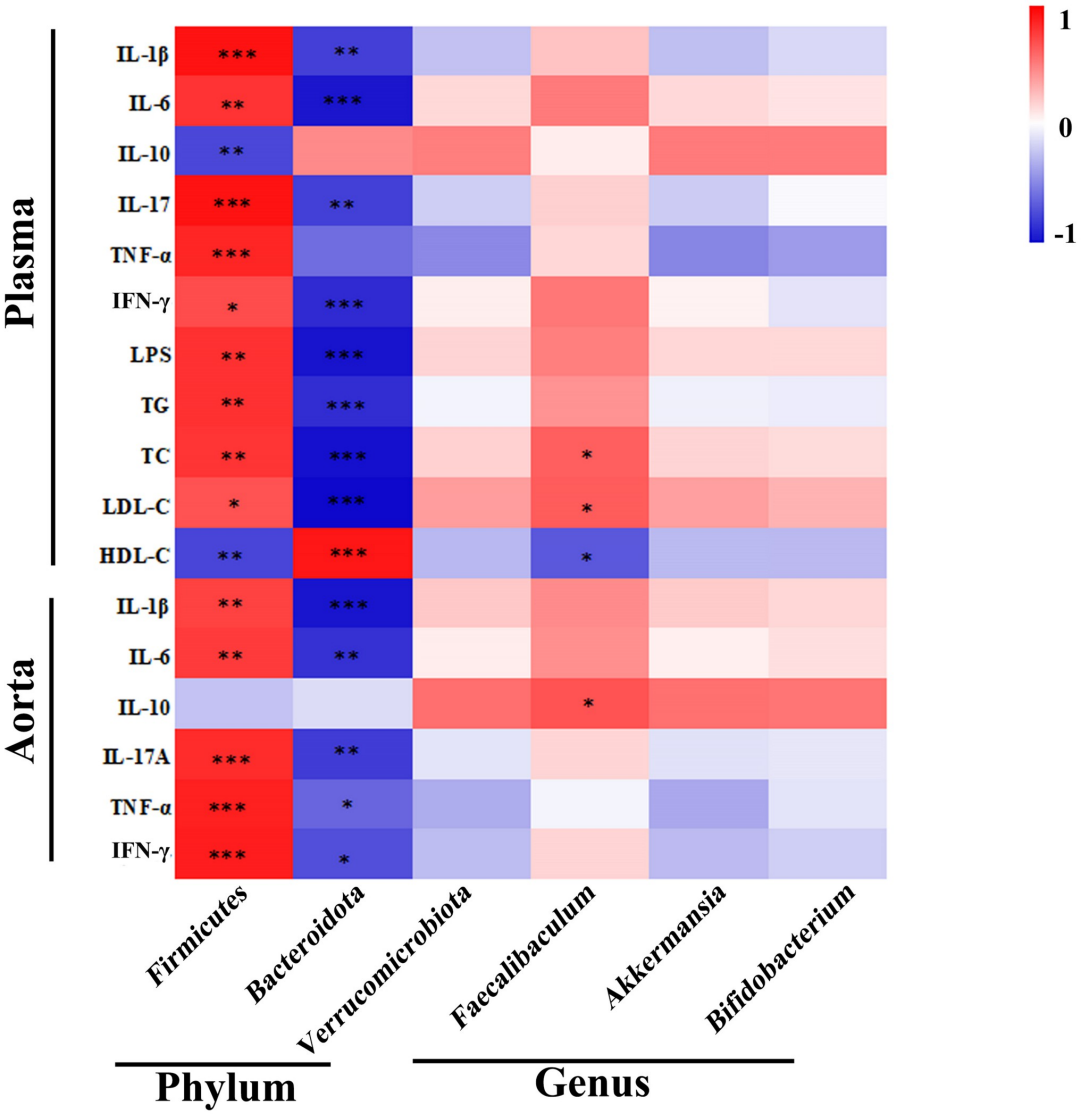
Altered gut microbiota in AS mice and their association with inflammatory indicators and serum lipids. The heat map showing the correlation of different microbial abundance with inflammatory indicators and serum lipids. The intensity of the color indicates the degree of correlation between the corresponding factor and each microbial species, which is obtained by Spearman’s correlation analysis. *P<0.05, **P<0.01, ***P<0.001.

For the assessment of interactions among the differential bacteria microbiota, inflammatory indicators, serum lipids in AS, correlation analysis was performed in AS and AS treated with NaB (Fig 7). In brief, the abundance of *Firmicutes* was found to be positively associated with the levels of pro-inflammatory indicators (TNF-α, IL-1β, IL-6, IL-17A, IFN-γ, LPS) and serum lipids (TG, TC, LDL-C), but negatively correlated with IL-10 and HDL-C. However, the beneficial bacteria *Bacteroidetes* were negatively correlated with metabolic and pro-inflammatory indicators (TG, TC, LDL-C, LPS, IL-1β, IL-17A, TNF-α, IFN-γ). The abundance of butyrate-producing bacteria *Faecalibaculum* exhibited a positive correlation with IL-10, whereas negatively correlated with TC and LDL-C. Taken together, there were close and complicated interactions among gut bacteria, inflammation, and serum lipids in AS and AS treated with NaB.

### NaB consumption notably modulated the aorta miRNAs

**Fig 8:**
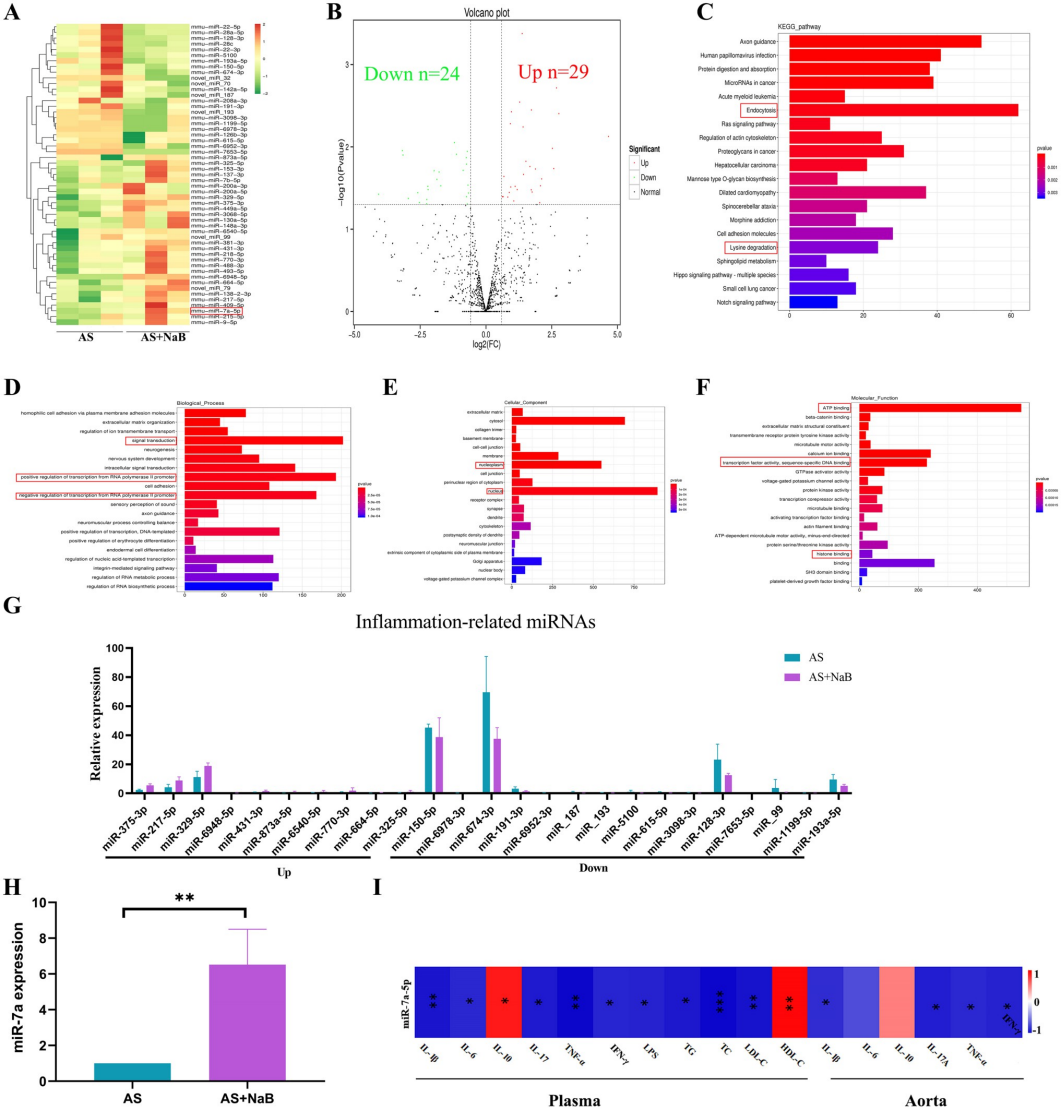
NaB altered the aorta transcriptome. (A) MiRNAs cluster heatmap with differential expression in AS and AS+NaB groups. (B) Differential-expression volcano diagram. (C) KEGG enrichment plot of differential miRNA host genes between AS and AS+NaB. (D-F) GO analysis of miRNA host genes between AS and AS+NaB. (G) Inflammation-related miRNAs. (H) The expression level of miR7a-5p was measured by real-time PCR. (I) Correlation analyses between the relative abundance of miR7a-5p and other related indicators. *P<0.05, **P<0.01, ***P<0.001. ns, no significant difference.

MicroRNAs (miRNAs) play an essential role in the regulation of atherosclerosis [24]. The cDNA and sRNA libraries of aortic tissue samples were sequenced. Moreover, counts of clean reads and mapped ratio of sequencing data were shown in Table 1. Under the NaB treatment, 53 miRNAs were identified to express differentially with the significance (P<0.05; Table 2). Compared with the AS group, up-regulated 29 miRNAs and down-regulated 24 miRNAs in the AS+NaB group were shown in the cluster heatmap (Fig 8A) and volcano diagram (Fig 8B). The most significantly enriched KEGG pathways were shown in Fig 8C. For the miRNAs, endocytosis was the most significantly enriched pathway. GO analysis contained the biological process (BP), cellular component (CC), and molecular function (MF) for host linear transcripts. Based on the GO enrichment analysis of the trans targeted genes of miRNA, the most significantly enriched BP, CC, and MF were signal transduction, nucleus, and ATP binding (Fig 8D–8F), respectively. It was indicated that atherosclerotic miRNAs were different with or without NaB treatment, suggesting that NaB may play an important role in atherosclerosis-related GO terms such as transcription factor activity. As shown in Fig 8G, compared with the AS group, a total of 25 inflammation-associated miRNAs were found. MiR-7a-5p was up-regulated after the supplementation with NaB in comparison with the AS group (P<0.05; Table 2). Increasing evidence supports that miR-7a-5p plays an important role in regulating the inflammatory process in inflammatory diseases [38]. MiR-7a-5p was identified as a protector of cardiac remodeling and hypoxia-induced injury in H9c2 cardiomyoblasts [26]. Subsequently, qRT-PCR of miR-7a-5p was identified in consistent with the RNA-sequencing results (P<0.01; Fig 8H). Moreover, we found that miR-7a-5p was negatively correlated with metabolic and pro-inflammatory indicators (TG, TC, LDL-C, TNF-α, IL-1β, IL-6, IL-17A, IFN-γ, LPS) and positively associated with HDL-C and IL-10 (Fig 8I). Taken together, the attenuation of dietary NaB on AS may be probably dependent on miRNAs regulation.

**Table 1:** Statistics on miRNAs sequencing data.

Sample ID		Raw_reads	Length<18	Length>30	Low_quality	Containing’N’reads	Clean_reads	Q30(%)
**AS**	AS-1	23,121,429	168,621	1,826,584	37	0	21,126,187	97.59
	AS-2	21,708,601	265,802	2,123,678	32	0	19,319,089	96.56
	AS-3	21,911,061	338,998	2,011,504	73	0	19,560,486	97.48
**AS+NaB**	AS+NaB-1	24,680,177	159,205	3,544,540	37	0	20,976,395	95.19
	AS+NaB-2	25,284,292	160,510	2,324,112	42	0	22,799,628	95.13
	AS+NaB-3	25,319,479	114,848	3,448,266	32	0	21,756,333	97.05

AS (AS-1, AS-2, AS-3): model group. AS+NaB (AS+NaB-1, AS+NaB-2, AS+NaB-3): NaB-fed model group.

**Table 2:** Differential miRNAs.

Name	AS-1	AS-2	AS-3	AS+NaB-1	AS+NaB-2	AS+NaB-3	*P* value	FDR	log2FC	Regulated
**mmu-miR-375-3p**	1.951506	2.380196	2.869506	6.50502612	5.739427628	4.47025643	0.000419	0.537216	1.375188	up
**mmu-miR-153-3p**	0.111515	0.250547	0.136643	0.23871655	1.947305802	0.71524103	0.001926	0.841813	2.683955	up
**mmu-miR-217-5p**	4.5721	2.067012	6.012297	6.92278009	11.68383481	7.98685816	0.002853	0.841813	1.281354	up
**mmu-miR-488-3p**	0.446059	0.876914	0.88818	1.19358277	3.689632047	1.43048206	0.003551	0.841813	1.726872	up
**mmu-miR-7a-5p**	168.5544	178.64	220.747	231.674416	445.2156003	310.235796	0.003749	0.841813	0.966961	up
**mmu-miR-137-3p**	0.223029	1.252735	0.409929	0.47743311	9.377814786	1.5496889	0.00394	0.841813	2.770577	up
**mmu-miR-329-5p**	6.85815	11.83834	14.82578	18.918287	16.75707888	20.9804035	0.005227	0.918867	0.927049	up
**mmu-miR-449a-5p**	0.390301	0.563731	0.956502	2.08876985	1.229877349	1.13246496	0.005734	0.918867	1.408897	up
**mmu-miR-6948-5p**	0	0	0	0.23871655	0.102489779	0.17881026	0.007472	1	4.655223	up
**mmu-miR-7b-5p**	1.505447	23.23823	3.962651	5.96791387	119.6055722	26.165901	0.010441	1	2.526312	up
**mmu-miR-431-3p**	0.223029	0.751641	0.819859	0.7758288	2.306020029	1.31127522	0.014992	1	1.518448	up
**mmu-miR-138-2-3p**	0.334544	0.125273	0.546572	0.35807483	1.537346686	0.83444787	0.017337	1	1.670665	up
**mmu-miR-873a-5p**	0.167272	0.18791	0	0.29839569	1.588591576	0.17881026	0.018343	1	2.578392	up
**mmu-miR-215-5p**	7.973296	6.827404	57.52675	14.3826724	148.9688939	22.8281095	0.018574	1	1.880817	up
**mmu-miR-200a-5p**	0.780602	2.129649	2.049647	7.93732545	2.613489367	1.37087864	0.021648	1	1.428764	up
**mmu-miR-381-3p**	276.8351	438.7703	463.8351	500.111182	615.9635723	585.067162	0.022945	1	0.683887	up
**mmu-miR-6540-5p**	0	0.313184	0.546572	0.17903742	2.152285361	0.59603419	0.02452	1	2.116643	up
**novel_miR_79**	0.111515	0.062637	0.068322	0.11935828	0.409959116	0.47682735	0.029691	1	2.071131	up
**mmu-miR-130a-5p**	0.83636	1.064825	0.819859	1.96941158	0.922408012	2.74175728	0.03015	1	1.109216	up
**mmu-miR-218-5p**	156.6223	357.4679	295.2175	266.407675	739.0537969	363.46165	0.030634	1	0.942004	up
**mmu-miR-493-5p**	0.61333	1.565918	1.434753	1.55165761	3.945856495	1.60929232	0.033052	1	1.18708	up
**mmu-miR-770-3p**	0.167272	0.939551	0.956502	0.41775397	4.099591163	1.07286154	0.033289	1	1.719991	up
**mmu-miR-664-5p**	0.167272	0.062637	0.136643	0.23871655	0.307469337	0.65563761	0.035307	1	1.753326	up
**mmu-miR-409-5p**	7.47148	7.954865	8.74516	9.01154994	20.29297626	10.3113915	0.035785	1	0.884158	up
**mmu-miR-148a-3p**	36460.54	40748.08	39247.25	59454.029	38542.92126	71473.3208	0.040312	1	0.625901	up
**mmu-miR-9-5p**	31.6144	39.8996	54.04235	46.907803	74.30508983	51.3781473	0.040323	1	0.652683	up
**mmu-miR-3068-5p**	2.118778	1.628555	3.142792	3.93882315	2.408509808	4.70867011	0.040844	1	0.828242	up
**mmu-miR-200a-3p**	10.92843	13.65481	37.44022	73.166624	19.47305802	14.6624411	0.045263	1	1.058341	up
**mmu-miR-325-5p**	0.111515	0.501094	0.136643	0.05967914	2.152285361	0.59603419	0.047837	1	2.045298	up
**mmu-miR-150-5p**	47.33796	42.59298	245.8893	42.7302633	23.9826083	49.6496481	0.008825	1	-1.19898	down
**mmu-miR-6978-3p**	0.278787	0.250547	0.204965	0	0	0.05960342	0.011124	1	-3.18015	down
**mmu-miR-674-3p**	54.08459	56.74888	98.04144	44.1625626	29.36332171	39.4574635	0.011323	1	-0.70937	down
**mmu-miR-191-3p**	1.895749	2.818653	4.645866	1.0742245	0.819918233	1.90730941	0.01228	1	-1.12206	down
**mmu-miR-6952-3p**	0.334544	0.125273	0.273286	0	0.05124489	0	0.012539	1	-3.15618	down
**mmu-miR-28a-5p**	73.5439	106.9209	189.6607	54.9644867	67.18205019	66.4578123	0.013497	1	-0.74735	down
**mmu-miR-22-5p**	51.8543	67.5224	144.7051	39.7463064	46.37662503	47.2059079	0.016183	1	-0.74109	down
**mmu-miR-28c**	3.847255	5.136212	9.223411	2.68556124	2.562244477	3.39739489	0.019308	1	-0.86962	down
**novel_miR_187**	0.223029	0.313184	1.503074	0.17903742	0.153734669	0.05960342	0.019505	1	-1.97054	down
**novel_miR_193**	0.446059	0.501094	0.614894	0.05967914	0.05124489	0.2980171	0.020169	1	-1.78143	down
**mmu-miR-5100**	0.167272	0.501094	2.322933	0.11935828	0.204979558	0.2980171	0.024805	1	-1.86558	down
**mmu-miR-22-3p**	1299.034	1377.82	3666.682	982.139585	1124.159142	1186.46566	0.02912	1	-0.68474	down
**novel_miR_70**	1.003632	0.37582	5.055795	0.53711225	0.768673343	0.11920684	0.029614	1	-1.71659	down
**mmu-miR-208a-3p**	0.446059	3.006563	0.204965	0.17903742	0.05124489	0.59603419	0.030218	1	-2.21246	down
**mmu-miR-615-5p**	0.055757	0.876914	0.956502	0	0.05124489	0.2980171	0.033092	1	-2.20113	down
**mmu-miR-3098-3p**	0.167272	0.062637	0.751537	0	0	0.11920684	0.035911	1	-2.59294	down
**mmu-miR-128-3p**	16.00235	18.10202	35.45889	11.8164695	12.04254904	14.0068035	0.036328	1	-0.66551	down
**mmu-miR-7653-5p**	0.111515	0.125273	0.136643	0	0	0	0.037648	1	-4.08196	down
**mmu-miR-142a-5p**	121.997	145.7557	433.6369	145.318703	109.8690432	86.9017851	0.040334	1	-0.74143	down
**novel_miR_32**	0.111515	0.062637	0.546572	0.05967914	0	0	0.042513	1	-2.94997	down
**mmu-miR-126b-3p**	0.167272	0.250547	0.273286	0	0.05124489	0.05960342	0.043583	1	-2.25026	down
**novel_miR_99**	0	0.313184	10.52152	0.05967914	0.358714227	0.89405129	0.04744	1	-2.53077	down
**mmu-miR-1199-5p**	0.167272	0.250547	0.273286	0	0	0.11920684	0.049041	1	-2.25542	down
**mmu-miR-193a-5p**	9.367229	6.451584	13.11774	4.29689799	5.175733843	6.25835901	0.04949	1	-0.70609	down

AS (AS-1, AS-2, AS-3): model group. AS+NaB (AS+NaB-1, AS+NaB-2, AS+NaB-3): NaB-fed model group.

## Discussion

In the present study, we investigated the efficacy of dietary NaB intervention on chronic AS. At the end of this experiment, we demonstrated that NaB could ameliorate the AS, as well as further revealed that the effectiveness was mainly attributed to the suppression of atherosclerotic inflammation by regulating Mψs polarization via GPR43/ HDAC-miRNAs axis in *ApoE*^*−*/−^ mice.

*ApoE*^-/-^ mice fed with HFD, a classical mouse model of AS, were used to test the hypothesis that NaB protect against AS. Since *ApoE*^-/-^ mice have a C57BL/6J genetic background, C57BL/6J mice were used as a CON group. Moreover, in parallel with previous studies, our results demonstrated that long-term NaB administration could ameliorate AS in mice [39, 40].

Dyslipidemias including hypercholesterolemia and hyperlipidemia, can further enhance the risk for atherosclerotic CVDs [41]. NaB has been thought to show a beneficial effect on the lipid metabolism in diverse chronic diseases [1]. Consistently, in this study, decreases of TG, LDL-C, TC levels, but an increase of HDL-C in AS with dietary NaB treatment demonstrated that NaB could attenuate lipid disturbance in AS development.

Mψs exerts a predominant inflammatory role in AS lesion formation as well as plaque rupture [42]. In the present study, the proportions of F4/80^+^ cells and F4/80^+^ TLR4^+^ cells were all significantly decreased with the dietary NaB administration, suggesting that the anti-inflammation effect of NaB was ascribed to the inhibition of inflammatory Mψs. Decreased levels of M1 iNOS, and elevated M2 CD206 indicated that NaB possessed the ability to ameliorate inflammation of AS via regulating Mψ M1/M2 polarization.

Concomitant with the reduction of pro-inflammatory Mψs, NaB administration also decreased the levels of pro-inflammatory cytokines (TNF-α, IL-1β, IL-6, IL-17A, IFN-γ), but elevated anti-inflammatory IL-10. A similar study found that combination of pro-inflammatory cytokines led to chronic inflammatory response in the arterial wall, which is thought to promote disease progression characterized by atherosclerotic plaque buildup [43]. Furthermore, the dramatic changes in Mψ described above may be responsible for the release of pro-inflammatory cytokines by LPS-TLR4-NF-κB/Nod 3-like receptor protein (NLRP3) inflammasome signaling [44]. However, whether other immune cells such as regulatory T cells (Tregs), Th17 cells and myeloid suppressor cells (MDSCs) are involved in the anti-inflammatory effects of the NaB treatment on AS still needs to be further explored.

After translocation into arteries, LPS links gut microbiota and pathogen-induced systemic inflammation, subsequently binds to TLR4 of Mψs, leading to an inflammatory cascade that ultimately aggravates AS [11]. Endotoxemia causes the activation of M1 Mψs, which promote the formation of AS foam cells [45]. Elevated plasma LPS level in AS indicated impaired gut barrier with abnormal integrity and permeability in consistent with previous reports [37, 46]. Importantly, this elevated plasma LPS of AS group was conversely changed by NaB intervention, suggesting that the effectiveness of NaB on chronic inflammation in AS was partially due to the reduction of LPS translocation.

Among the numerous pathogenesis in AS, gut dysbiosis is increasingly thought to be critical in the inflammation of AS [47]. We found that NaB could notably change gut microbial composition by improving anti-inflammatory related bacteria (*Bacteroidetes*, *Verrucomicrobiota*, *Akkermansia*, *Bifidobacterium*) and butyrate-producing bacteria (*Faecalibaculum*), but decreasing *Firmicutes* and F/B ratio, suggesting that the protection of NaB against the inflammation of AS may partially attribute to the rectification of gut dysbiosis. *Akkermansia* and *Bifidobacterium* are conductive to the reduction of LPS leakage via protecting the gut mucosal barrier function [48, 49]. *Bifidobacterium* plays a synergistic effect in the improvement of inflammation to further alleviated the atherosclerosis [50]. *Akkermansia* is implicated in declining aortic lesions and atherosclerosis [51]. *Akkermansia* can also stimulate goblet cells to secret mucus and elevate the expression of gut junction proteins [52]. In addition to these microbiota, butyrate-producing bacteria *Faecalibaculum* is also lack in atherosclerotic CVD [53].

Positive correlations of increased *Firmicutes* with metabolic indicators (TG, TC, LDL-C) demonstrated that these pathogenic bacteria were related to the lipid dysmetabolism of AS. In contrast, *Bacteroidetes* were negatively correlated with these indicators. These findings indicated that gut microbiota may play crucial role in etiology of dyslipidemia. However, the underlying mechanisms of gut microbiota affects blood lipid levels remains unclear. It has been addressed that gut bacteria could generate SCFAs, modulating hepatic and/or systemic lipid and glucose metabolism via the activation of nuclear or GPCRs [54, 55]. Meanwhile, gut microbiota also modulate the metabolism of bile acids which is the main end-product of cholesterol [56].

Gut microbiota is closely associated with the integrity and permeability of gut barrier which is indicated by the tight junction proteins [53, 57]. In our study, the improvement of tight junction protein ZO-1 revealed that oral NaB intervention may contribute to the attenuation of the integrity of gut barrier, thereby reducing LPS translocation and ultimately suppressing atherosclerotic chronic inflammation.

Apart from the above restorement of NaB on gut dysbosis-related inflammation, NaB also directly serve as a novel anti-inflammation approach by binding to the GPR43 [16, 58]. Thus, we speculated and proved that NaB regulated inflammatory Mψs and their polarization through GPR43-β-arrestin-2-mediated pathways by increasing the levels of M2 (CD206, IL-10 and PPARγ) and reducing M1 indicators (iNOS, TNF-α and NF-κB/NLRP3).

As an inhibitor of HDACs, NaB can regulate the inflammation through the acetylation regulation of inflammatory gene expression [59]. In the present study, LPS significantly increased the accumulation of HDAC1-3/Sp1 and reduced PPARγ acetylation in Mψs. However, dietary NaB restored PPARγ acetylation and expression, PPARγ further repressed pro-inflammatory NF-κB /NLRP3 pathways. In parallel with our study, Saemann et al. has demonstrated that butyrate could inhibit the secretion of TNF-α and the activation of NF-κB and up-regulate the expression of anti-inflammatory factors IL-10 in LPS-activated mononuclear cells and neutrophils via HDAC inhibition [60]. Moreover, butyrate also increases the expression of PPARγ, which act as an E3 ubiquitin ligase of NF-κB/p65 to promote its degradation [61]. Here, we demonstrated that NaB may prevent atherosclerotic chronic inflammation through the HDAC/Sp1/PPARγ/NF-κB or NLRP3 signaling pathway, but the exact mechanism still needs to be further investigated in vitro experiment.

MiRNAs have emerged as evolutionarily conserved, noncoding small RNAs that serve as important regulators and fine-tuners of a range of pathophysiological cellular effects and molecular signaling pathways involved in atherosclerosis [62]. In recent years, there has been increased interesting in the role of miRNAs on macrophage polarization which mainly rely on the regulation of vital signaling pathways [63, 64]. Inhibition of miRNA-155 attenuates AS via reducing M1 Mψ polarization and inflammatory responses in mice [64]. MiRNA-130a suppression can protect against atherosclerosis through inhibiting inflammation by regulating the PPARγ/NF-κB expression [65]. In addition, miRNAs are closely related to HDACs in different human chronic diseases and cancerogenic pathways [66]. To date, many miRNAs have been found directly targeted by HDACs in chronic metabolic diseases [67–69]. Intriguingly, in our study, 25 differential inflammation-related miRNAs candidates were found by transcriptomic analysis after long-term dietary NaB supplementation. Especially, miR-7a-5p was identified and proved to be closely interacted with the inflammation. Due to the complex relationships about roles of above differential miRNAs in AS with NaB treatment, the underlying mechanisms need to be further investigated.

## Conclusion

**Fig 9:**
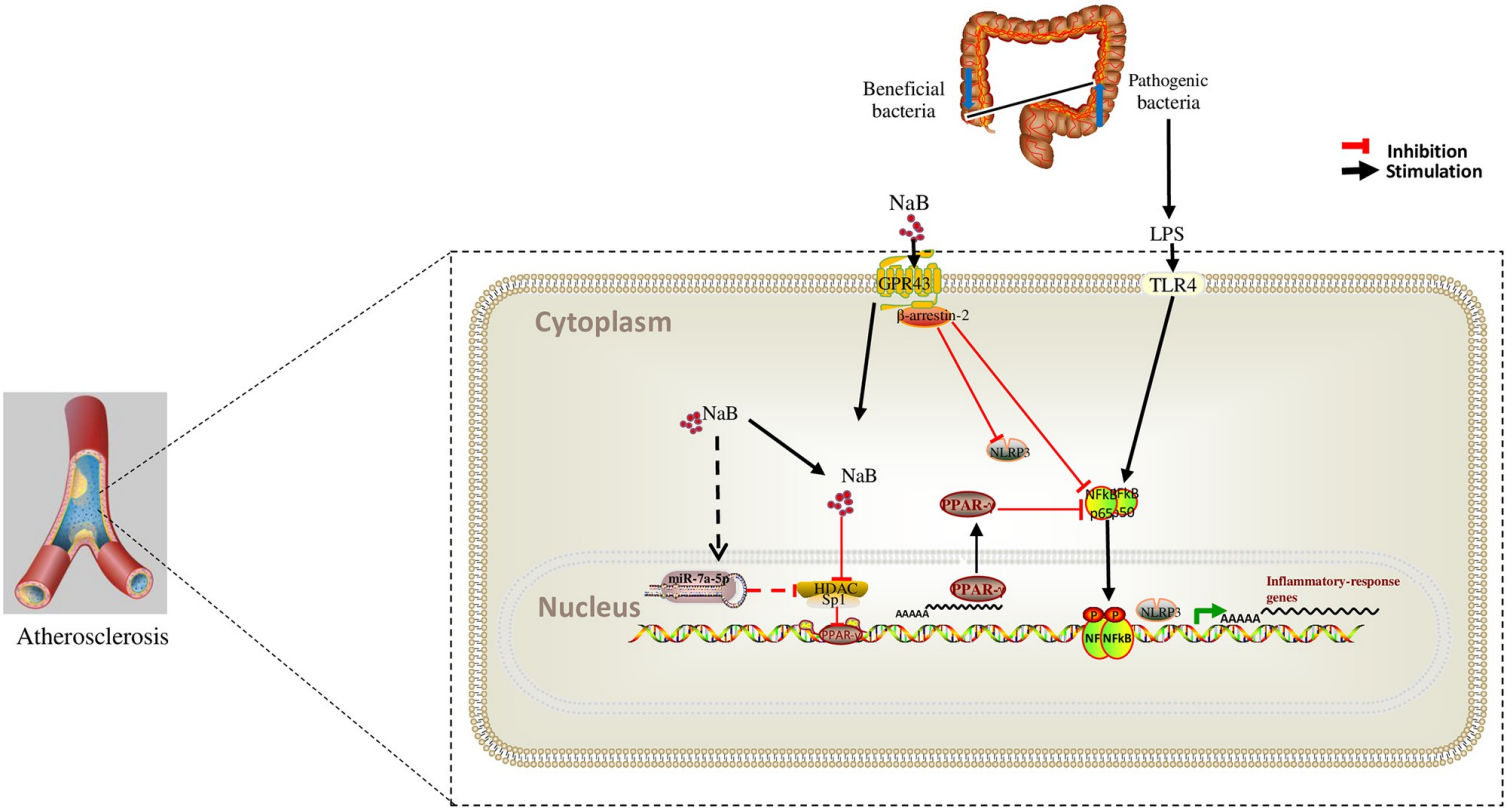
Schematic diagram of anti-inflammation effect of NaB in AS.

Our study provides a new evidence that butyrate could ameliorate the progression of inflammation in atherosclerosis through regulating macrophage polarization via GPR43-related and HDAC/PPAR-γ/NF-κB/NLRP3/miRNAs signal pathways. Schematic mechanism is shown in Fig 9.

## Supporting information

S1 Dataset (DOCX)

S1 Table Ingredients of high fat diet and normal diet. (DOC)

S2 Table Primer sequences of above genes and miRNAs. (XLSX)
