# Supplementary material for: Butyrate suppresses atherosclerotic inflammation by regulating macrophages and polarization via GPR43/HDAC-miRNAs axis in ApoE−/− mice
Source: PLoS One. 2023 Mar 8;18(3):e0282685. doi: 10.1371/journal.pone.0282685 (PMC9994734; doi:10.1371/journal.pone.0282685)
Supplement: S1 Dataset — (DOCX) [file pone.0282685.s001.docx]

1. Data for Fig. 1C-G.

| Groups | Food intake | TG | TC | HDL-C | LDL-C |
| --- | --- | --- | --- | --- | --- |
| CON | 34.27±0.15 | 0.43±0.08 | 2.76±0.27 | 2.49±0.02 | 2.91±0.14 |
| CON+NaB | 35.07±0.22 | 0.38±0.05 | 2.17±0.20 | 2.21±0.08 | 2.48±0.09 |
| AS | 35.43±0.24 | 3.30±0.23 | 9.10±0.37 | 0.84±0.05 | 11.42±0.12 |
| AS+NaB | 36.87±0.35 | 0.76±0.02 | 5.73±0.63 | 1.43±0.09 | 8.54±0.75 |

2. Data for Fig. 2B-E

| Groups | Aorta lesion area(%of total aorta) | Oil red O staining (%) | Masson’s trichrome staining(%) | Necrotic core area(%) |
| --- | --- | --- | --- | --- |
| CON | 0.21±0.06 | 0.31±0.12 | 7.38±0.44 | 0.29±0.09 |
| CON+NaB | 0.22±0.05 | 0.15±0.09 | 8.00±1.34 | 0.28±011 |
| AS | 26.23±0.92 | 18.06±2.80 | 20.67±1.82 | 17.39±0.73 |
| AS+NaB | 20.67±0.88 | 6.83±0.79 | 8.71±1.15 | 12.83±0.90 |

3. Data for Fig. 3A-C

| Groups | IL-1β | IL-6 | IL-10 | IL-17A | TNF-α | IFN-γ |
| --- | --- | --- | --- | --- | --- | --- |
| CON | 1426±21.9 | 1906±102.5 | 4045±320.1 | 1191±83.21 | 1605±33.81 | 3872±114.5 |
| CON+NaB | 1398±59.25 | 2059±120.9 | 4711±69.26 | 1605±32.49 | 1276±47.86 | 2038±35.23 |
| AS | 7038±88.01 | 10634±597.1 | 2682±81.39 | 5784±646.5 | 2083±36.53 | 6759±553.7 |
| AS+NaB | 1602±53.46 | 5270±549.3 | 5241±150.2 | 1782±54.13 | 1225±98.79 | 4048±374.8 |

| Groups | *IL-1β* | *IL-6* | *IL-10* | *IL-17A* | *TNF-α* | *IFN-γ* | LPS |
| --- | --- | --- | --- | --- | --- | --- | --- |
| CON | 1.00±0.00 | 1.00±0.00 | 1.00±0.00 | 1.00±0.00 | 1.00±0.00 | 1.00±0.00 | 0.18±0.01 |
| CON+NaB | 0.48±0.19 | 1.42±0.44 | 0.91±0.35 | 0.311±0.16 | 1.06±0.26 | 0.54±0.10 | 0.17±0.01 |
| AS | 4.15±0.55 | 20.69±4.65 | 0.54±0.24 | 3.021±0.25 | 6.65±0.88 | 3.58±0.80 | 3.10±0.10 |
| AS+NaB | 1.44±0.47 | 9.443±1.05 | 3.40±0.78 | 0.851±0.37 | 0.83±0.28 | 0.49±0.15 | 0.55±0.03 |

4. Data for Fig. 4B, C, E, G

| Groups | F4/80^+^ cells (%) | F4/80^+^ TLR4^+^cells (%) | F4/80^+^cells (%) | | F4/80^+^iNOS^+^cells(%) | | F4/80^+^ CD206^+^cells (%) | |
| --- | --- | --- | --- | --- | --- | --- | --- | --- |
| CON | 1.22±0.06 | 0.23±0.02 | | 1.22±0.06 | | 0.90±0.02 | | 0.62±0.11 |
| CON+NaB | 1.18±0.06 | 0.21±0.01 | | 1.18±0.06 | | 1.01±0.29 | | 0.71±0.14 |
| AS | 6.13±0.35 | 1.40±0.25 | | 6.13±0.35 | | 2.19±0.42 | | 0.35±0.04 |
| AS+NaB | 3.73±0.17 | 0.55±0.05 | | 3.73±0.17 | | 0.56±0.25 | | 1.34±0.40 |

5. Data for Fig. 5A-I

| Groups | *HDAC1* | *HDAC2* | *HDAC3* | *SP1* | *PPAR--γ* | *GPR43* | *β-arrestine2* | *NF-κB* | *NLRP3* |
| --- | --- | --- | --- | --- | --- | --- | --- | --- | --- |
| CON | 1.00±0.00 | 1.00±0.00 | 1.00±0.00 | 1.00±0.00 | 1.00±0.00 | 1.00±0.00 | 1.00±0.00 | 1.00±0.00 | 1.00±0.00 |
| CON+NaB | 0.69±0.07 | 0.69 ±0.01 | 0.66±0.05 | 0.64±0.13 | 0.95±0.11 | 0.59±0.04 | 0.65±0.38 | 0.84±0.16 | 0.91±0.19 |
| AS | 2.18±0.27 | 2.14±0.16 | 4.87±0.73 | 3.52±0.22 | 0.52±0.11 | 0.91±0.10 | 1.14±0.32 | 3.62±0.31 | 5.79±1.22 |
| AS+NaB | 0.63±0.12 | 0.87±0.13 | 1.44±0.59 | 0.69±0.06 | 3.41±0.29 | 3.02±0.47 | 3.61±0.21 | 0.86±0.35 | 0.34±0.11 |

6. Data for Fig.6C-J

| Groups | *Firmicutes* | *Bacteroidetes* | *Firmicutes/Bacteroidetes* | *Verrucomicrobiota* | *Akkermansia* | *Bifidobacterium* |
| --- | --- | --- | --- | --- | --- | --- |
| CON | 0.44±0.05 | 0.35±0.03 | 1.25±0.04 | 0.00±0.00 | 0.00±0.00 | 0.01±0.00 |
| CON+NaB | 0.44±0.02 | 0.39±0.03 | 1.08±0.11 | 0.01±0.00 | 0.01±0.00 | 0.03±0.00 |
| AS | 0.73±0.02 | 0.05±0.01 | 19.96±6.71 | 0.12±0.00 | 0.1±0.00 | 0.02±0.01 |
| AS+NaB | 0.44±0.05 | 0.15±0.04 | 3.33±0.78 | 0.21±0.05 | 0.21±0.05 | 0.04±0.01 |

| Groups | *Faecalibaculum* | *ZO-1* |
| --- | --- | --- |
| CON | 0.01±0.00 | 1.00±0.00 |
| CON+NaB | 0.01±0.00 | 0.53±0.09 |
| AS | 0.06±0.03 | 0.47±0.04 |
| AS+NaB | 0.27±0.07 | 3.25±0.14 |

7. Data for Fig.8H

| Groups | miR-7a-5p |
| --- | --- |
| AS | 1.00±0.00 |
| AS+NaB | 6.53±1.14 |
