# Supplementary material for: Butyrate suppresses atherosclerotic inflammation by regulating macrophages and polarization via GPR43/HDAC-miRNAs axis in ApoE−/− mice
Source: PLoS One. 2023 Mar 8;18(3):e0282685. doi: 10.1371/journal.pone.0282685 (PMC9994734; doi:10.1371/journal.pone.0282685)
Supplement: S1 Table — (DOC) [file pone.0282685.s002.doc]

**S2 Table1. Ingredients of high fat diet and normal diet**

| **Ingredient（%）** | Normal diet | High fat diet |
| --- | --- | --- |
| Caisein | 7.5 | 7.5 |
| Soy Protein | 13 | 13 |
| DL-Methionine | 0.2 | 0.2 |
| Cornstarch | 42.22 | 26.25 |
| Dyetrose | 13 | 13 |
| Surcose | 3 | 2 |
| Cellulose | 9 | 9 |
| Soybean Oil | 5 | 5 |
| Cocoa Butter | - | 16 |
| Mineral Mix | 3.5 | 3.5 |
| Calcium Carbonate | 0.55 | 0.55 |
| Sodium Chloride | 0.8 | 0.8 |
| Potassium Citrate H2O | 1 | 1 |
| Vitamin Mix | 1 | 1 |
| Choline Bitartrate | 0.2 | 0.2 |
| Cholestrol | 0.03 | 0.5 |
| Cholate | - | 0.5 |
